# Functional Characterization of *TaSnRK2.8* Promoter in Response to Abiotic Stresses by Deletion Analysis in Transgenic *Arabidopsis*

**DOI:** 10.3389/fpls.2017.01198

**Published:** 2017-07-13

**Authors:** Hongying Zhang, Ruilian Jing, Xinguo Mao

**Affiliations:** ^1^College of Tobacco Science, Henan Agricultural University Zhengzhou, China; ^2^The National Key Facility for Crop Gene Resources and Genetic Improvement, Institute of Crop Science, Chinese Academy of Agricultural Sciences Beijing, China

**Keywords:** *TaSnRK2.8*, inducible promoter, abiotic stress, abscisic acid, deletion analysis

## Abstract

Drought, salinity, and cold are the major factors limiting wheat quality and productivity; it is thus highly desirable to characterize the abiotic-stress-inducible promoters suitable for the genetic improvement of plant resistance. The sucrose non-fermenting 1-related protein kinase 2 (*SnRK2*) family genes show distinct regulatory properties in response to abiotic stresses. The present study characterized the approximately 3000-bp upstream sequence (the 313 bp upstream of the ATG was the transcription start site) of the *Triticum aestivum TaSnRK2.8* promoter under abscisic acid (ABA) and abiotic stresses. Four different-length 5′ deletion fragments of *TaSnRK2.8* promoter were fused with the *GUS* reporter gene and transformed into *Arabidopsis*. Tissue expression analysis showed that the *TaSnRK2.8* promoter region from position -1481 to -821 contained the stalk-specific elements, and the region from position -2631 to -1481 contained the leaf- and root-specific elements. In the ABA-treated seedlings, the deletion analysis showed that the *TaSnRK2.8* promoter region from position -821 to -2631 contained ABA response elements. The abiotic stress responses of the *TaSnRK2.8* promoter derivatives demonstrated that they harbored abiotic-stress response elements: the region from position -821 to -408 harbored the osmotic-stress response elements, whereas the region from position -2631 to -1481 contained the positive regulatory motifs and the region from position -1481 to -821 contained the leaf- and stalk-specific enhancers. Further deletion analysis of the promoter region from position -821 to -408 indicated that a 125-bp region from position -693 to -568 was required to induce an osmotic-stress response. These results contribute to a better understanding of the molecular mechanisms of *TaSnRK2.8* in response to abiotic stresses, and the *TaSnRK2.8* promoter seems to be a candidate for regulating the expression of abiotic stress response genes in transgenic plants.

## Introduction

Plant growth is adversely affected by abiotic stresses such as salinity, drought, and low temperature. To cope with environmental stresses, plants have developed intricate signaling networks to protect their cellular activities and maintain whole-plant integrity. Increasing evidence indicates that the *SnRK2* (sucrose non-fermenting 1-related protein kinase 2) family plays a crucial role in response to environmental stresses (Boudsocq et al., 2004, 2007; Kobayashi et al., 2004).

The *SnRK2* family is a plant-specific gene family that encodes serine/threonine kinases. It is divided into three distinct subclasses (I, II, and III) based on their functional divergence (Halford and Hardie, 1998). In *Arabidopsis*, 10 *SnRK2* members were cloned. Of these, five members (*AtSnRK2.2*, *AtSnRK2.3*, *AtSnRK2.6*, *AtSnRK2.7*, and *AtSnRK2.8*) were activated by abscisic acid (ABA). All members, except *AtSnRK2.9*, responded to drought and salt stresses, and none was induced by cold stress (Boudsocq et al., 2004, 2007). Similarly, in rice, these 10 *SnRK2* members, named *SAPK1–10*, were induced by osmotic stresses, and three (*SAPK8–10*) were also induced by ABA treatment (Kobayashi et al., 2004). In maize, 10 *ZmSnRK2* genes were activated by one or more abiotic stresses (Huai et al., 2008). In wheat, the first identified *SnRK2*-family gene, *PKABA1*, was isolated from an ABA-treated embryo cDNA library (Anderberg and Walker-Simmons, 1992). In our recent study, 10 wheat *SnRK2* genes were identified, and all responded to multiple stressors (Zhang et al., 2016). Among these, *TaSnRK2.1–3* (subclass II) responded weakly to ABA, *TaSnRK2.4–7* (subclass I) were not induced by ABA treatment, and *TaSnRK2.8–10* (subclass III) were strongly activated by ABA (Zhang et al., 2016). Thus, the *SnRK2* members from subclasses I and III have distinct stress signals. Functional analysis of *SnRK2* genes indicated that the overexpression of *TaSnRK2.3*, *TaSnRK2.4*, *TaSnRK2.7*, or *TaSnRK2.8* in *Arabidopsis* led to enhanced tolerance to abiotic stresses (Mao et al., 2009; Zhang et al., 2010, 2011; Tian et al., 2013). Various studies have shown that the ABA-activated *SnRK2* genes are involved in stress responses through ABA signaling (Melcher et al., 2009; Nishimura et al., 2009; Sheard and Zheng, 2009). However, to date, little research has been conducted on the molecular mechanisms of SnRK2s in response to abiotic stress.

To improve stress tolerance in plants, a large number of genes have been expressed under the control of stress-inducible or constitutive promoters. Constitutive promoters, including the rice *Act1* (McElroy et al., 1990), cauliflower mosaic virus *CaMV35S* (Odell et al., 1985), and maize *Ubi1* promoters (Christensen et al., 1992), have been widely used in plant biotechnology applications. However, the high and constitutive expression of transgenes might impose an extra metabolic burden and other adverse effects on plants, finally resulting in their abnormal development (Shelton et al., 2002). Thus, organ-specific and inducible promoters in plant cells have considerable potential for use in plant genetic engineering. Some abiotic-stress-inducible promoters, including *Atrd29A* (Yamaguchi-Shinozaki et al., 1990), *OsABA2* (Rai et al., 2009), and *OsNAC6* (Checker et al., 2012), have been characterized in plants. In this study, we characterized and analyzed the wheat *TaSnRK2.8* promoter (subclass III) in *Arabidopsis*. A series of different-length *TaSnRK2.8* promoters were fused to the *GUS* gene to detect the putative regulatory *cis*-elements that could confer tissue-specific expression and induce stress signaling in *Arabidopsis*.

## Materials and Methods

### Plant Materials

For the evolutionary analysis of the *TaSnRK2.8* promoter sequences, 10 wheat species were used: hexaploid wheat (*Triticum aestivum*, AABBDD) cultivars “Hanxuan 10” and “Chinese Spring”; tetraploid wheat (AABB) PS9 (*Triticum persicum*) and DM 50 (*Triticum dicoccum*); diploid wheat *Triticum urartu* (AA) UR201 and UR204; diploid wheat *Aegilops speltoides* (BB) Y2001 and Y2041; and diploid wheat *Aegilops tauschii* (DD) Y85 and Ae46. Leaves were harvested for DNA isolation.

### Sequence Isolation and Analysis

To isolate *TaSnRK2.8* promoter, its genomic sequence (Zhang et al., 2013) was used as a query to screen the Wheat Genome Browser^1^. The approximately 3000-bp upstream sequence of the translation start site was considered to be the putative *TaSnRK2.8* promoter region. It was polymerase chain reaction (PCR)-amplified using the primers listed in supplementary Supplementary Table S1.

The *SnRK2.8* promoter sequences from wheat (MF351624), rice (LOC_Os03g55600), and maize (AC206916.1) were aligned for element prediction using MegAlign in the DNAStar software, and then predicted using the PlantCARE software^2^. To gain insight into the evolution and origin of the TaSnRK2.8 promoter sequences, the hexaploid wheat varieties and their relatives were used to construct a phylogenetic tree using MEGA 7.0.

The transcriptional start site was determined by 5′ rapid amplification of cDNA ends (RACE) using the GeneRacer Kit (Invitrogen, China). Total RNA was isolated using TRIzol reagent (Sangon, China). Total RNA (3.5 μg) was used as a template for 5′-RACE. The gene-specific primers used for the nested PCR are listed in Supplementary Table S1. The nested PCR products were cloned into the pGEM-T-Easy vectors and sequenced.

### Transgenic Plant Generation

**FIGURE 1:**
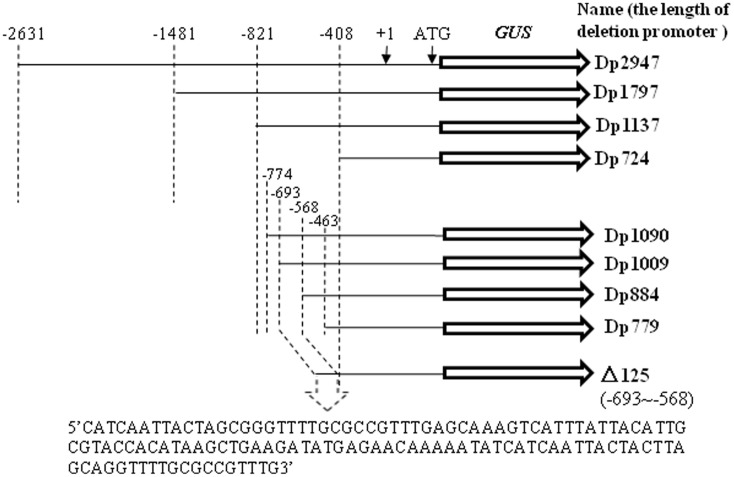
Schematic depiction of deletion fragments generated in the study. Successive 5′ promoter truncations were fused to a *GUS* reporter. The putative transcription start site was designated as “+1”.

Successive 5′ truncations of the *TaSnRK2.8* promoter were PCR-amplified using the primers shown in Supplementary Table S1. Nine promoter fragments (-2631 to ATG, Dp2947; -1481 to ATG, Dp1797; -821 to ATG, Dp1137; -774 to ATG, Dp1090; -693 to ATG, Dp1009; -568 to ATG, Dp884; -463 to ATG, Dp779; -408 to ATG, Dp724; Dp724 carrying the 125-bp fragment from position -693 to -568, named as Δ125) were separately fused with the *GUS* reporter gene in pCAMBIA1391, yielding the P*_TaSnRK2_*:*GUS* constructs (**Figure 1**). These expression constructs and the pCAMBIA1391 vector (CaMV35S:GUS) were transformed into *Agrobacterium*, and then transferred into the wild-type *Arabidopsis* plants by floral infiltration. The transgenic plants were initially screened on hygromycin plates and subsequently characterized by PCR (Supplementary Table S1) and *GUS* (beta-glucuronidase) staining. The T_3_-generation plants were selected for subsequent functional analyses.

### Quantitative Real-Time PCR analysis

Ten similarly sized 10-day-old seedlings from each transgenic line were used for total RNA isolation using the TRIzol reagent (Sangon) and then treated with RNase-free DNase (Takara, China). The quantitative real-time PCR (qRT-PCR) assays were performed in triplicate with the SYBR Premix Ex Taq (Takara, Shiga, Japan) using an ABI PRISM 7000 system (Applied Biosystems, Foster City, CA, United States). The relative transcript levels of *GUS* gene were determined using the 2^-ΔΔCT^ method (Livak and Schmittgen, 2001). The *actin* transcript was used to quantify the expression level.

### Southern Blotting

Total genomic DNA was extracted from 10 similarly sized 10-day-old seedlings collected from each transgenic line using the Qiagen DNeasy Mini Kit (Qiagen, Valencia, CA, United States). Approximately 10 μg DNA was digested with *Eco*RI, segregated in 0.8% agarose gels, and then blotted onto nylon membranes (Hybond N+; Amersham Biosciences, Little Chalfont, United Kingdom). A 993-bp *GUS* construct labeled with digoxigenin (DIG) was used as a probe. Probe hybridization was performed using the AlkPhos Direct Labeling and Detection System (Amersham Biosciences) according to the manufacturer’s protocol.

### Abiotic Stress Treatments

Seeds were germinated on Murashige and Skoog (MS) medium solidified with 1.0% agar, and cultured in a growth chamber (25°C, 14 h light/10 h dark cycle, and 150 μMM^-2^ S^-1^ light intensity). After 7 days, the seedlings were planted on MS medium containing 150 mM mannitol, 150 mM NaCl, or 50 μM ABA, which were shown to constitute a significant stress in our pilot experiments. In cold stress treatment, seedlings were planted on MS medium and cultured in a cold growth chamber (4°C). Control plants were grown under non-stress conditions. After 3 days, twenty 10-day-old seedlings of each transgenic line were collected and subjected to *GUS* activity assays. All experiments were performed in triplicate.

### *GUS* Activity Assays

Histochemical and fluorometric analyses of the *GUS* activity were performed as described previously (Jefferson, 1987). Seedlings were incubated in *GUS* reaction buffer at 37°C for 24 h, and then rinsed in an ethanol series to remove chlorophyll. Tissue observations were performed using a Leica MZ12 binocular microscope and images were captured using a Leica DC300 camera (Leica Microsystems, Wetzlar, Germany).

For the fluorometric assay, plants were homogenized in *GUS* extraction buffer. The homogenate was centrifuged at 12,000 × *g* for 10 min at 4°C, and then the supernatant activity was detected via an assay buffer containing 1 mM 4-methylumbelliferyl-β-glucuronide (Sigma, United States) at 37°C. The reaction was terminated by the addition of 200 mM Na_2_CO_3_ to a final concentration of 180 mM. Fluorometric estimations were performed using a PerkinElmer LS45 spectrofluorimeter (PerkinElmer).

### Statistical Analysis

The Student’s *t*-test, variance analysis, and Chi-square test, were performed using the statistical software SAS 9.2 to determine significant differences (*P* < 0.05) between the means.

## Results

### Regulatory Element Analysis

The putative transcription start site was determined by two independent 5′-RACE experiments, indicating that the nucleotide position 313 bp upstream of the ATG start codon was the putative transcription start site of *TaSnRK2.8* (Supplementary Figure S1).

The possible *cis*-elements were predicted by the multiple alignments of the *SnRK2.8* promoter sequences from wheat, rice, and maize, and then determined using the PlantCARE software. As shown in **Table 1**, 11 predicted elements, including one ABA response element (ABRE), two binding sites for MYB transcription factors, and one low-temperature response element (LTRE), were identified in the *TaSnRK2.8* promoter.

**Table 1:** Predictions of *cis*-elements present in the *TaSnRK2.8* promoter.

N	Element name	Conserved sequence/strand	Start position
1	ABRE	CACGTG(+)	-1165
2	MBS	GTCAAT(-) TAACTG(+)	-2539 -1576
3	LTR	CCGAAA(+) CCGAAA(+)	-393 +24
4	Unknown	CTACTCC(+)	-11
5	Unknown	AAAAAAT(+)	-2462, -2375
6	Unknown	AATTTT(+)	-2645, -1188
7	Unknown	AATCTA(+)	-2661, -2650, -2124, -1586, -1260, -903, -264
8	Unknown	ATGTAT(+)	-2165, -2134, -1753, -1648, -305, -274
9	Unknown	TGTATC(+)	-2145, -285
10	Unknown	TTTTTAGGCA(+)	-80
11	Unknown	CATTTATGAC(+)	+7


*Possible cis-elements were predicted by multiple alignments using the SnRK2.8 promoter sequences from wheat, rice, and maize*.

### Evolutionary Analysis of the *TaSnRK2.8* Promoter

**FIGURE 2:**
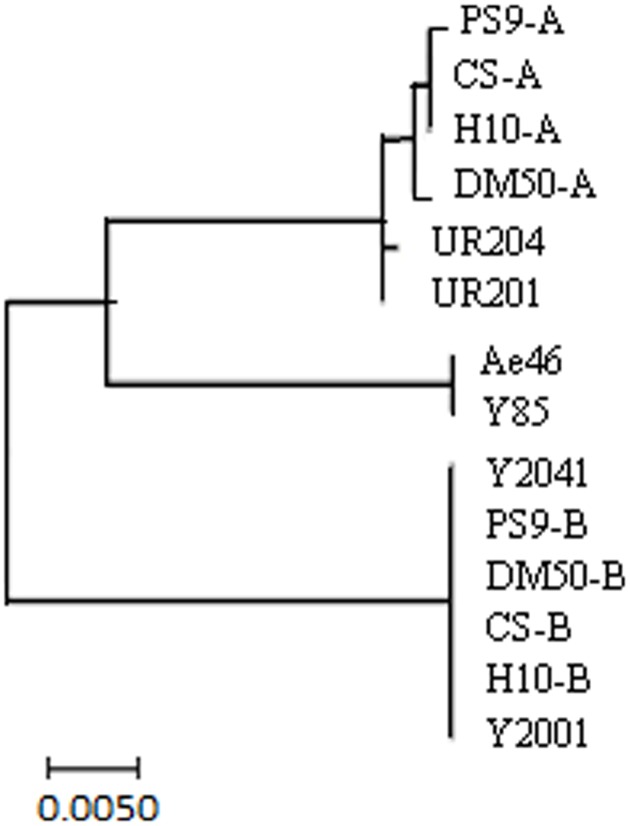
Phylogenetic analyses of *TaSnRK2.8* promoters from hexaploid, tetraploid, and diploid wheats. Hexaploid wheat: Chinese Spring (CS) and Hanxuan 10 (H10); tetraploid wheat: PS9 and DM 50; A genome diploid: UR201 and UR204; B genome diploid: Y2001 and Y2041; D genome diploid: Y85 and Ae46. Phylogenetic analysis was conducted in MEGA 7.0.

The hexaploid wheat *T. aestivum* originated from hybridization between the diploid goat grass (*Ae. tauschii*, DD) and the tetraploid emmer wheat (*Triticum turgidum*, AABB) (Dubcovsky and Dvorak, 2007). To gain insight into the origin of the *TaSnRK2.8* promoter sequences, the hexaploid wheat varieties and their relatives were used to construct a phylogenetic tree. Consistent with the genomic sequence analysis of *TaSnRK2.8* (Zhang et al., 2010), these *TaSnRK2.8* promoter sequences were divided into three distinct groups. As shown in **Figure 2**, two genotypes (*P_TaSnRK2.8_-A* and *P_TaSnRK2.8_-B*) were present in all hexaploid and tetraploid wheats: *P_TaSnRK2.8_-A* and *P_TaSnRK2.8_-B* originated from the A and B diploids, respectively. No *TaSnRK2.8* promoters were detected in the D diploids. Moreover, no genetic diversity of *TaSnRK2.8* promoters existed in the B and D genomes. The *TaSnRK2.8* promoter in B genome was further examined through the *GUS* activity assays.

### Identification of Transgenic Plants

**FIGURE 3:**
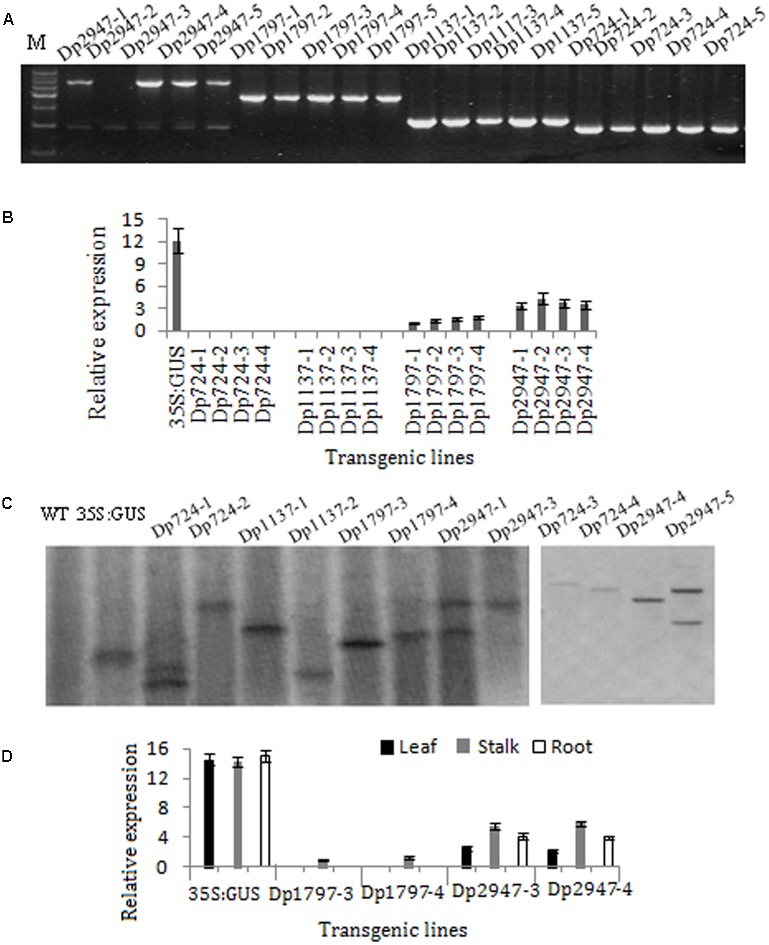
Identification of transformed *Arabidopsis* plants. **(A)** DNA-PCR analysis of transgenic plants. **(B,D)** Expression levels of *GUS* under the control of *TaSnRK2.8* promoter deletion segments. Values are means ± SD (*n* = 3). **(C)** Southern blot analysis of transgenic plants. Total genomic DNA from the transgenic *Arabidopsis* leaves was digested with *Eco*RI. A DIG-labeled DNA fragment of *GUS* was used as the hybridization probe.

To characterize the function of the *TaSnRK2.8* promoter, a series of 5′ promoter deletions were performed and four derivatives of *TaSnRK2.8* promoter fragments were fused to the *GUS* (beta-glucuronidase) reporter gene (**Figure 1**). Hygromycin segregation in the T_1_ and T_2_ seeds was analyzed through the Chi-square tests. The T_3_ homozygous lines were used for performing the *GUS* activities assays. The integration of DNA in transformed plants was identified via PCR (**Figure 3A**). The qRT-PCR performed to determine the transcript levels of *GUS* under non-stress conditions showed that different promoter constructs have different abilities to regulate gene expression, and no *GUS* transcription was detected in the Dp724 and Dp1137 plants (**Figure 3B**). Southern blotting was used for selecting two independent transgenic lines with a single gene copy, which were further used in the *GUS* activity assays (**Figure 3C**). Expression patterns of *GUS* in various tissues showed that *GUS* was strongly expressed in all tissues of 35S:GUS seedlings, weakly in Dp2947 seedlings, and marginally in seedling stalks of Dp1797 plants under non-stress conditions. For Dp2947 plants, the transcript levels of *GUS* in stalks and roots were higher than those in leaves (**Figure 3D**).

### Deletion Analysis of the *TaSnRK2.8* Promoter in Transgenic Seedlings under Non-stress Conditions

**FIGURE 4:**
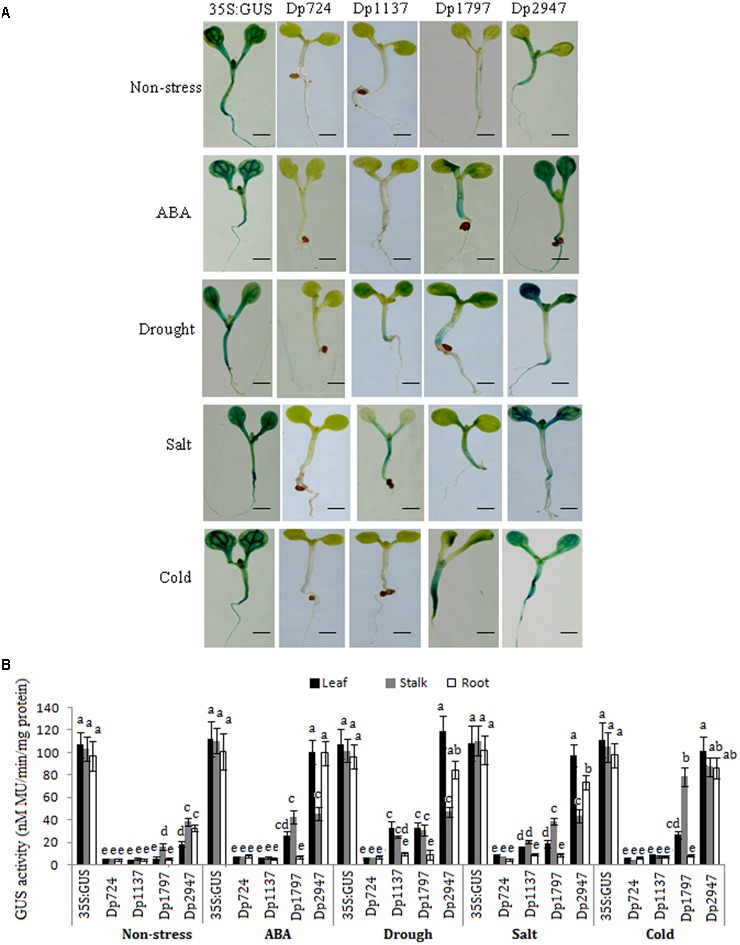
Effects of various abiotic stresses on the *GUS* activity under the control of different *TaSnRK2.8* promoter deletion segments in the transgenic *Arabidopsis* seedlings. **(A)** *GUS* staining of transgenic *Arabidopsis* seedlings containing promoter deletion constructs under normal and stress conditions. **(B)** *GUS* activity under the control of different *TaSnRK2.8* promoter deletion segments. The 7-day-old seedlings were planted on the MS medium containing 150 mM NaCl (salt), 150 mM mannitol (drought), or 50 μM ABA. Cold, cultured in a cold growth chamber (4°C). The plants used for the analysis were two independent lines carrying the promoter deletion constructs. *GUS* activity was expressed as nM MU/min/mg protein. Values are the mean ± SE (*n* = 20). ^a-e^Values followed by the different letter are significantly different (*P* < 0.05). Dp724, Dp1137, Dp1797, and Dp2947 indicate the *TaSnRK2.8* promoter regions from –408 to +316, –821 to +316, –1481 to +316, and –2631 to +316, respectively. Bar, 2 mm.

Under non-stress conditions, the CaMV35S plants displayed the highest *GUS* expression intensity in all tissues, whereas only a slight *GUS* expression was observed in the controls of the *TaSnRK2.8* promoters (Dp2947). Moreover, different expression patterns and activities were observed among the deletion segments of the *TaSnRK2.8* promoter(**Figure 4**).

No *GUS* activity was observed in the Dp724 and Dp1137 plants, and only a faint expression was detected in the stalks of the Dp1797 seedlings under non-stress conditions. Although Dp2947 induced *GUS* expression in all tissues, weak GUS activity was observed in the leaves. These results indicated that the 660-bp segment (from position -1481 to -821) might harbor the stalk-specific expression elements under non-stress conditions, and that some enhancers were conserved in the region from position -2631 to -1481.

### Activity of the Promoter Fragments in Response to ABA and Abiotic Stresses

Under ABA treatment, the *GUS* expression was detected in Dp1797, but not in Dp724 and Dp1137 plants. When the promoter length was increased to position -2631, stronger *GUS* activities were detected in the leaves and roots of the Dp2947 seedlings (**Figure 4**). This finding suggested that the region from position -821 to -2631 might harbor some ABREs.

In the abiotic stress assays, no *GUS* activity was observed in the Dp724 (-408) seedlings. When the promoter length was increased to position -821 (Dp1137), a weak *GUS* expression was observed in the leaves and stalks in response to drought and salt stresses (**Figure 4**), suggesting the likely presence of osmotic stress-response elements in the region from position -821 to -408. The Dp1797 (-1481) seedlings showed relatively higher *GUS* expression levels in the leaves and stalks under various stresses, whereas no *GUS* activity was observed in the roots. Under stress treatments, the longest promoter, Dp2947, conferred the highest levels of *GUS* expression in all tissues, predominantly in the leaves. The results indicated that the fragment from position -1481 to -821 contained positive regulatory elements that enhanced gene expression in the leaves and stalks. Furthermore, the *TaSnRK2.8* promoter (Dp2947) conferred the highest gene expression, suggesting the likely presence of positive regulatory motifs in the region from position -2631 to -1481.

### A 125-bp Region was Required for Osmotic Stress Response

**FIGURE 5:**
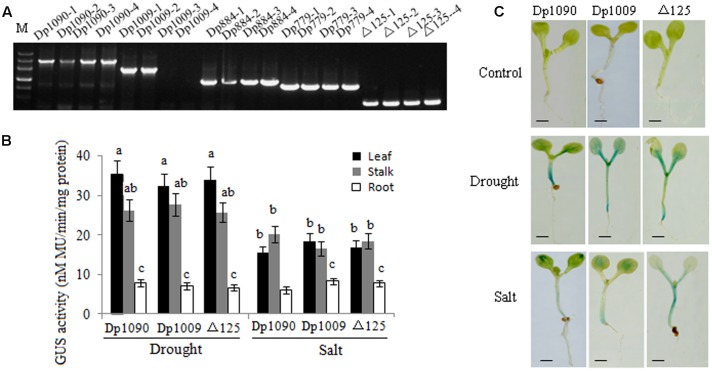
*TaSnRK2.8* promoter region from –693 to –568 (125 bp) is required for osmotic stress response. **(A)** DNA-PCR analysis of transgenic plants. **(B)** Effects of osmotic stress on *GUS* activity under the control of different *TaSnRK2.8* promoter deletion segments. The plants used for the analysis were two independent lines carrying the promoter deletion constructs. *GUS* activity was expressed as nM MU/min/mg protein. Values are the mean ± SE (*n* = 20). ^a–c^Values followed by the different letter are significantly different (*P* < 0.05). **(C)** The *GUS* staining of transgenic *Arabidopsis* seedlings containing promoter deletion constructs under normal and osmotic stress conditions. The 7-day-old seedlings were planted on the MS medium containing 150 mM NaCl (salt) or 150 mM mannitol (drought). –774 to ATG, Dp1090; –693 to ATG, Dp1009; –568 to ATG, Dp884; –463 to ATG, Dp779; –408 to ATG, Dp724; Dp724 carrying the 125-bp fragment from –693 to –568 bp, Δ125. Bar, 2 mm.

The promoter region from position -821 to -408 was used for further deletion analysis (**Figure 2**). The integration of DNA in the transformed plants was identified via PCR (**Figure 5A**). Under drought and salt stresses, no *GUS* activity was observed in the Dp779 and Dp884 seedlings. Increasing the length of the promoter to position -693 (Dp1009) or -774 (Dp1090) led to the detection of *GUS* activity in the leaves and stalks in response to osmotic stress in a pattern similar to the Dp1137 seedlings (**Figures 5B,C**). This finding suggested that some osmotic stress-response elements might exist in the 125-bp region from position -639 to -568. To further verify the osmotic stress response element, the 125-bp region was fused to Dp724 through an overlapping PCR, which cannot induce the *GUS* transcription under non-stress and stress conditions. It was found that the promoter fragment Dp724 carrying the 125-bp region could induce the expression of *GUS* in the leaves and stalks in response to osmotic stress, very similar to what was observed in the Dp1009, Dp1090, and Dp1137 seedlings (**Figures 5B,C**). Collectively, these results suggest that the 125-bp *TaSnRK2.8* promoter region from position -693 to -568 was required for inducing an osmotic stress response in *Arabidopsis* plants. The effects of different *TaSnRK2.8* promoter deletion segments in response to ABA and abiotic stresses were shown in Supplementary Figure S2.

## Discussion

### Different Promoter Fragments of *TaSnRK2.8* Harbored Different Elements for Tissue-Preferential Expression

Previous studies found that *TaSnRK2.8* (subclass III) was strongly expressed in the roots, and faintly in the stems and leaves. The over-expression of *TaSnRK2.8* in *Arabidopsis* enhanced its tolerance to abiotic stresses through different stress-signaling routes (Zhang et al., 2010, 2011, 2016). To more thoroughly investigate the signaling and regulation of *TaSnRK2.8* under different stresses, we characterized the spatial expression pattern of different *TaSnRK2.8* promoter derivatives under ABA and environmental stress conditions.

Gene expression patterns might be a direct indication of a promoter’s function in stress and developmental processes. In *Arabidopsis*, the *GUS* gene driven by *AtSnRK2.7* promoter was specifically expressed in the roots, whereas the *AtSnRK2.8* promoter-mediated *GUS* activity was observed in all tissues (Umezawa et al., 2004; Mizoguchi et al., 2010). Furthermore, *AtSnRK2.6* was confirmed to play a key role in stomatal closure under stress conditions (Mustilli et al., 2002). These data indicate that the SnRK2 family members play different roles in different tissues. The promoter deletion analyses of *TaSnRK2.8* indicated that different promoter fragments harbored different elements for tissue-preferential expression. In general, the region from position -1481 to -821 contained the stalk-specific elements, whereas the region from position -2631 to -1481 contained the leaf- and root-specific elements. The spatial variation in expression levels was closely related to the different physiological stages of the plant lines and to the duration of stress treatment.

### *TaSnRK2.8* Promoter Could Drive Strong Gene Expression in Response to ABA

ABA is a vital hormone involved in plant abiotic stress responses. Plants can respond to stresses via ABA-dependent or -independent pathways. As a stress hormone, ABA acts through the regulatory pathways that control gene expression in response to stress. Increasing evidence shows that the subclass III SnRK2s respond strongly to ABA, the subclass II SnRK2s are only faintly induced by ABA, and the subclass I SnRK2s are moderately induced by ABA (Kobayashi et al., 2004, 2005; Boudsocq et al., 2007; Huai et al., 2008; Fujita et al., 2009). In the present study, the ability of *TaSnRK2.8* promoter to induce *GUS* expression in *Arabidopsis* revealed that *TaSnRK2.8* could drive transgene expression in response to ABA, and the promoter region from position -821 to -2631 might contain ABREs.

### *TaSnRK2.8* Promoters Harbored Abiotic-Stress Response Elements

The identification of tissue-specific or inducible promoters in expression analysis would be of considerable practical value because it could eliminate the unnecessary burdens on plants by restricting the genetic expression to specific tissues or in response to specific environmental conditions (Hsieh et al., 2002; Zavallo et al., 2010; Hou et al., 2016). Here, we examined the activation of *GUS* under the regulation of *TaSnRK2.8* promoter fragments in response to abiotic stresses and showed that a typical stress-inducible promoter contains some regions for regulating tissue specificity and transcription levels under stress, leading to different regulation patterns. For example, there might be osmotic stress response elements in the region from position -693 to -568 and positive regulatory motifs from -2631 to -1481, whereas the fragment from -1481 to -821 contained the leaf- and stalk-specific enhancers. Further analysis might enable the identification of essential control elements in these functional regions.

The comparative functional analysis of different stress-responsive promoters has provided useful information, leading to enhancements in the specificity and inducibility of stress responsive genes. Therefore, the *TaSnRK2.8* promoter examined in the present study might prove beneficial for the overexpression of foreign genes in the plants subjected to abiotic stresses. Moreover, the findings of the deletion analysis reported here provide the basis for identifying the direct upstream regulators of SnRKs and stress-response elements in *TaSnRK2.8* promoter.

## Author Contributions

HZ performed the experiments and participated to the data analysis. XM performed the qRT-PCR experiments, RJ projected design and supervision.

## Conflict of Interest Statement

The authors declare that the research was conducted in the absence of any commercial or financial relationships that could be construed as a potential conflict of interest.

## Footnotes

1 http://www.ebi.ac.uk/ena/search/

2 http://bioinformatics.psb.ugent.be/webtools/plantcare/html/
