# Supplementary material for: Functional Characterization of TaSnRK2.8 Promoter in Response to Abiotic Stresses by Deletion Analysis in Transgenic Arabidopsis
Source: Front Plant Sci. 2017 Jul 13;8:1198. doi: 10.3389/fpls.2017.01198 (PMC5507967; doi:10.3389/fpls.2017.01198)
Supplement: Supplementary file 1 [file Table_1.DOCX]

**Functional Characterization of TaSnRK2.8 Promoter in Response to Abiotic Stresses by Deletion Analysis in Transgenic *Arabidopsis***

**Runing title:** Characterization of *TaSnRK2.8* promoter

Hongying Zhang^1,2,†,*^, Ruilian Jing^2,†^, Xinguo Mao^2,†^

^1^ College of Tobacco Science, Henan Agricultural University, Zhengzhou 450002, China

^2^ The National Key Facility for Crop Gene Resources and Genetic Improvement, Institute of Crop Science, Chinese Academy of Agricultural Sciences, Beijing 100081, China

^†^ The authors contributed equally to this work

^*^ Corresponding author: Hongying Zhang

Email: [zhangying198215@163.com](mailto:zhangying198215@163.com)

Tel/Fax: 86-10-63555763

ATAATATAATCTACTGCTAATCTAATTTTGGTCAAAAGGTTCCATAATGTAGAGTAAGCGAACGAAAGAGAGAAAGAAAATGAGACTACATTGTGCGGCCGGTGCCTTTGCATGACATGCTGCTAGCATCAGTTACTAGTGGGTTTTGTGTTGTCTGAGCAAAGTTATTTATTACATTGCGTACCACATAAGCTAAATATATGAGAAAAAAATCATACGTTACTAGCGGGTTTTGCGCCGTTTGAGCAGATTCATATATTACATTGTGTATAACATAAGCTAAAGACATGAGAAAAAAATATCATCCATTACTAGCGGATTTTGCGCCGTTTGAGCAAAGCCATCTATTACATTGTGCATCAGATAAGCTAAATATATGAGATATATGCAGAAAACTATTATATAATAACATCTGATGCGCCGGAAATCATATCTTATTTTCAAGTGTAAAATGAATTGGTCACATACATAGTTAGGAAAGCATATCATGAAACAATATTTACATGTATAGTAATATCCATTTTGTATCATACAATGTATAAATAATCTAATGTTGGTCAAGGATAACAAAAATAAAGAATAATGCCGGAAATAGCAAAATGGACAGCAGAAGAGTAAATTGTGCGCTTGGTGCCTTTGCACAACACGGTACTGCCCGTTACTAGTGGGCTTTGTGCAGTTCCAGCCCAATCATTTATACATTTTGTGTTGTATAAGCTAAAGACATGACAAAATATATATATATGAAAAACAATTATAGAATAATATTTTGTGCACAGCTGGCTTGTTCACAACAAGCCATCTGTGGTGGTCAAAAGGAGAGTAAATAGTGTGGTGGGTGCCTTTTAGGAGCAGGGTGCAACCACTCGTTACCAGCAGGTTTTGAGCTATTCTATCCAAGTCATTTATGATATTATGTATTGCAAAACTTAATATACGCCAAAGATAATATATGGAAGACCATTATATAATAATATCTTTTGCATCGAAAACCATACACCATTACTCTGTTGTTTCCGCATGTATAAAATGGCTTGTTCACAAACATAGAAAACAAGAACATAAATGTTTTCTATGAGTATAATCTACTGCTAACTGCATGTTGGCCAAGGATGCCAAAATGAAGAACAAGGGAAGGCAATAGGAAAGTAAGAACATAATAAGAGAAAATTGTTAACATGTACCTTTGTGACTTCGTGTTGCCGTTTGTTACTAGCGGGTATCGTGCCATTTGATCCAAGGCATTCATTGCATATGTCATAAGCTAAAGAGACAATGTTTTTTTGCCAAAAACCATACCTCATTTTTGTTGTTGTTTATTCAAGTATATAAGTTGACTTGTTCACATGCATAGTTAGAAAAAGAGAACATGAAAGAAATATCGGCAAACATAACAATAGCATCATATAATCTACTAATAGTTATTGGTCAAAGATGCCAAAATGAAGAATAAGTGAAGGAACTAGAAAAATCAGATGAAAATTTTTAGAGTAAAATGCTCGGCACGTGAATTTGTGTGACATAGTGCTACCGTCCGTTACTAGTGAGTGTTGTGTCATTTGCGCCTAGTCACTATTACATTGCGTATGGCATAAGCTAATGATACGAGAAAATAATCATGAGTGACCATTATACAACAATATCTTTTGCACCAAAAATCATACCTCATATTGTTGTTGTTGTTATTTTTTTCCAAGTATAAGTTGATTTGTTCACATACATAGTTAGAAAATTAGAACATTTACAAATATCATAACATCATAATAATCTACTGCTAATCTAATGTTGGTCAAAAGGTTCCAAAATGTAGAGTAAGCGAACAAAAGAGAGAAAGAAAAATGAGACTGGATTGTGCGGGCGGTGCCTTTGCATGACATGGTGCTACCATCCGTTACTAGTGTTTTTTTTGTCGTTTGAGCATAGTTATTTATTACATTGCGTACCACATAAGCTGAAGATATGAGAACAAAAATATCATCAATTACTAGCGGGTTTTGCGCCGTTTGAGCAAAGTCATTTATTACATTGCGTACCACATAAGCTGAAGATATGAGAACAAAAATATCATCAATTACTACTTAGCAGGTTTTGCGCCGTTTGAGCAAATCCATTAATTCATTGCGTAGAACATAAGCTAAAGACATGAGAAAGAAATACCATCCGTTACTAGCGGATTTTGCGCCGTTTGAGCAAAGCCATTTATTACATTGTGCATCAGATAAGCTAAAGATATGAGATATATACAGAAAACCATTATACAATTATATCCTATGCGCCGAAAATCATATCTTATTTTTTTTCTAGCATAAGATGAATTGCTCACATACATAGTTAGAAAAACATATCATGAGAACAATATTTACATGTATAGCAGCATCCATTTTGTATCATACAATGTATAAATAATCTAATGTTGGTCAAAGATAACAAAAGCAAAAAACAATGAAGTAGAAAAATGGACAAAAGAATAGCGGAGATACATGTTTTTTTTTGCCTGTCAAGAGAGGATTCTGCATAGGTTGCCATCTTATTTGCTGTATTTCATGCTTAATGCTCCTATTAAAATGCAGATATACTCTTAGTAGCTTTTTTTAGGCAGTGTAACATGAGGGCTCAACAAACTTCCATATATATCCAGTGGAAAACCATGGGGAACCCTACTCCTCCCCCACATCATTTATGACACAAGTGCCGAAAGGTGACGTCCAATAAAACTACTTCCCAGAGGAGCGCTTTGCACCAAGCACCAACCCCGGCAATAATAAGACGGGCGAGAGAGTGATTGAGCGGGCGTTTTATCTTTCTCTCTCTCTCTCGGTCGGCCAGTGTCACGAACTCGTCGCGGCGGAGGGGAGAGAGGGGGGCTGAGCTCGCCGTCGATTCGAGGGGAGAGGAGGCGGGAAGAGAGGGGCGGCCACCCCGGGGGACCGAACCCTATCGGCCGCGGCCCCCCCTCCATCGACCGCCAGTCGCCGCCGGCCATG

**Figure S1. The *TaSnRK2.8* promoter used in the study.** The putative transcription start site was determined by RACE PCR. The putative transcription start site and the start codon are shown in red.


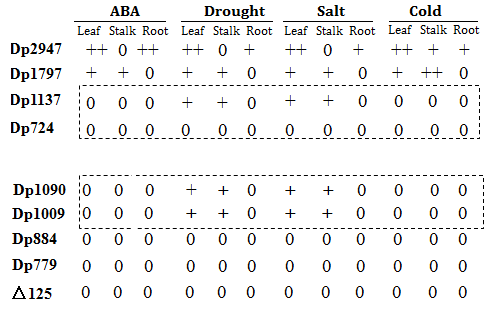


**Figure S2.** **Effects of various abiotic stresses on GUS activity under the control of different *TaSnRK2.8* promoter deletion segments in transgenic *Arabidopsis* seedlings.** For each construct, the relative staining intensities and GUS activities in leaves (L), stalks (S) and roots (R) in response to ABA and abiotic stresses are given. 0, none; +, significantly higher than non-stress seedlings at *P*﹤0.05; ++, significantly higher than non-stress seedlings at *P*﹤0.01.

**Table S1.** Primers used for sequence analysis

| Primer name | Sequence (5'→3') | Function |
| --- | --- | --- |
| P_TaSnRK2.8_-F | GGTTCCATAATGTAGAGTAAG | promoter isolation of TaSnRK2.8 |
| P_TaSnRK2.8_-R | GAGCCGATGTCCCGCA | promoter isolation of TaSnRK2.8 |
| P_TaSnRK2.8_-1 | GGGGAGGCAAACAGGCT | The first nested PCR in 5' RACE |
| P_TaSnRK2.8_-2 | TGTACTTGACGGCGACGAG | The second nested PCR in 5' RACE |
| T8-1-F  T8-2-F  T8-3-F  T8-4-F  T8-5-F  T8-6-F  T8-7-F  T8-8-F  T8-R  QGUS-F  QGUS-R  GUS-F  GUG-R | cgcGAATTCGGTTCCATAATGTAGAGTAAG  cgcGAATTCTGTGACTTCGTGTTGCCGT  cgcGAATTCGATTGTGCGGGCGGTG  cgcGAATTCATTATATCCTATGCGCCGAAAA  cgcGAATTCCTAGTGTTTTTTTTGTCGTTTGAGCA  cgcGAATTCCATCAATTACTAGCGGGTTTTG  cgcGAATTCAGCAAATCCATTAATTCATTGCG  cgcGAATTCCATTGTGCATCAGATAAGCTA  acgcGTCGACCGCTGTCGTGCATGATCG  ACACCGACATGTGGAGTGAA  TCATTGTTTGCCTCCCTGCT  gcggatcaacaggtggt  CACTCCACATGTCGGTGT | TaSnRK2.8 promoter truncation (-2,631 to +392 bp)  TaSnRK2.8 promoter truncation (-1,481 to +392 bp)  TaSnRK2.8 promoter truncation (-821 to +392 bp)  TaSnRK2.8 promoter truncation (-408 to +392 bp)  TaSnRK2.8 promoter truncation (-774 to +392 bp)  TaSnRK2.8 promoter truncation (-693 to +392 bp), △125  TaSnRK2.8 promoter truncation (-568 to +392 bp)  TaSnRK2.8 promoter truncation (-463 to +392 bp)  TaSnRK2.8 promoter truncation  QPCR analysis of *GUS*  QPCR analysis of *GUS*, transgenic lines detection with T8-F  Southern blot analysis  Southern blot analysis |
